# Is Ambient PM_2.5_ Sulfate Harmful? Schwartz and Lepeule Respond

**DOI:** 10.1289/ehp.1205873R

**Published:** 2012-12-03

**Authors:** Joel Schwartz, Johanna Lepeule

**Affiliations:** Department of Environmental Health, Harvard School of Public Health, Boston, Massachusetts, E-mail: jschwrtz@hsph.harvard.edu

Grahame and Schlesinger make two arguments against the conclusions of our paper (Lepeule et al. 2012). Regarding their first point, we argued that if sulfates are non-toxic—and the fraction of particles that are sulfate has declined substantially since the 1970s—one would expect a slope to have increased over time because the non-toxic fraction of PM (particulate matter) mass was declining. Grahame and Schlesinger make no concrete argument against it, other than to say that it conflicts with the approaches we advocated in another paper (Mostofsky et al. 2012). This is not true.

To see this, let us put our argument more mathematically. Consider the model

log(hazard ratio) = *b*_0_ + *b*_1_(*t*)PM_2.5_, [1]

where *b*_0_ is the baseline hazard and *b*_1_(*t*) is the possibly time varying slope of PM_2.5_. We said that if sulfates (SO_4_) had no toxicity and the ratio changed over time, we would expect

*b*_1_(*t*) = *c*_1_ + *c*_2_(SO_4_/PM_2.5_)*_t_,* [2]

and because *b*_1_(*t*) has no time trend, *c*_2_ is zero. By substitution we obtain

log(hazard ratio)= *b*_0_ +*c*_1_PM_2.5_ + *c*_2_SO_4_, [3]

which is precisely the model that we advocated in Mostofsky et al. (2012). If *c*_2_ is zero, then sulfates are neither more nor less toxic than average, which is the conclusion we drew in our paper (Lepeule et al. 2012).

Regarding the second point, we believe that the toxicity of ammonium sulfate per se misses the more general point: What does the addition of acidic sulfates into the atmosphere do to produce particles that are toxic? A typical process involves the sulfur dioxide (SO_2_) emissions from a coal-burning power plant being converted into sulfuric acid. This acid—or products formed from it—coats the outside of other particles (or adsorbs them), such as metal oxide particles from, for example, brake wear of cars and trucks, from tire wear, or from metal processing. Through internal mixing, the surface components diffuse into the inside of the particles; the acidic sulfates react with the metals, converting insoluble (and hence low toxicity) metal oxides into metal ions that are readily soluble in the lung lining fluid. This is critical because transition metals can cata--lyti-cally induce the production of highly reactive oxygenating compounds.

Ghio et al. (1999) reported that soluble iron concentrations correlate with sulfate concentrations in particles, and that the ability of soluble extracts from the particles to generate damaging oxidants was directly proportional to the sulfate concentrations. Rubasinghege et al. (2010) simulated the transformation of non-bioavailable iron to dissolved iron in atmospheric iron particles in the presence of acids, and found that the presence of sulfuric acid on the particles resulted in a dramatic increase in the bioavailable iron.

Transformations of metal particles are not the only way sulfates transform particles. Elemental carbon particles undergo chemi-cal modification over time. Popovicheva et al. (2011) showed that the extent of water uptake and modification of elemental carbon particles depended on the sulfate content of the particles. In addition, Li et al. (2011) reported that sulfate aided the aging of freshly emitted soot particles.

It is clear that sulfates contribute to the formation of secondary organic particles. For example, Wu et al. (2007) examined the effect of ammonium sulfate aerosol on the photo-chemical reactions of toluene (mostly from cars) and nitrogen oxides to form those secondary organic particles. They found that the sulfate particles reduced the time to reach maximum concentrations of secondary organic aerosols and also increased the total aerosol yield from toluene. That is, in the presence of sulfates, more gaseous emissions from mobile sources were converted into particles.

Hence there is good reason to believe that SO_2_ emissions and consequent sulfate particles in the air cause an increase in the number and toxicity of particles in the air that is proportional to the amount of sulfate, which makes the many epidemiologic findings of toxicity associated with sulfate quite plausible.
